# Slow change blindness from serial dependence: slowly changing objects are perceived as biased toward the past

**DOI:** 10.1186/s12915-026-02700-w

**Published:** 2026-08-03

**Authors:** Haley G. Frey, David Whitney

**Affiliations:** 1 Herbert Wertheim School of Optometry and Vision Science Program, University of California, Berkeley, Berkeley, CA 94720 USA; 2 Department of Psychology, University of California, Berkeley, Berkeley, CA 94720 USA; 3 Helen Wills Neuroscience Institute, University of California, Berkeley, Berkeley, CA 94720 USA

**Keywords:** Visual stability, Active visual perception, Temporal integration, One-shot trials, Color change

## Abstract

**Background:**

Slow change blindness, when attentive observers fail to notice large changes that happen gradually, raises questions about how visual information is combined across time. One plausible integration strategy is serial dependence: blending information from the recent past into current perception. We investigate serial dependencies in perception of a cartoon object that slowly changes hue. In a one-shot experiment, observers each viewed a single trial with a random degree of hue change and provided one hue judgment response. Across participants, the entire morph was probed.

**Results:**

Observers’ hue reports revealed an overall bias toward the past that increased in magnitude as more of the morph was experienced. In three follow-up experiments, we verified that observers experienced slow change blindness, confirmed that the bias was serial dependence, and replicated the results with a repeated-trials design.

**Conclusions:**

Overall, we provide evidence that serial dependence actively biases perception during gradual changes, sometimes producing slow change blindness.

**Supplementary Information:**

The online version contains supplementary material available at https://doi.org/10.1186/s12915-026-02700-w.

## Background

Attentive observers fail to notice otherwise obvious changes when the change takes place gradually, a phenomenon called slow change blindness [1]. Such changes include the appearance or disappearance of an object [1], rotation in scene orientation [2], and drift in the color or hue [1, 3, 4]. Slow change blindness persists even when the change region is large, occurs in full view, and is centrally located [4] and surprisingly, even when the change involves highly relevant features such as facial expression [3] or age [5].

This striking inability to notice large changes raises questions about how perception is generated across time. Most proposed explanations of slow change blindness are borrowed from classic change blindness paradigms (e.g., flicker paradigm [6]; mud-splash paradigm [7]) and revolve around the notion that there are distinct pre- and post-change stimulus states. Change blindness, they propose, results from (a) a failure to encode one or both states (implicit updating hypothesis [2]; world as external memory [8]) or (b) a failure to compare the contents of the two encoded states [8–10]. Yet comparing two scenes or objects is a fundamentally different task for the visual system than continuous viewing during which there are no clear pre- and post-change states. Therefore, while classic change blindness may result from insufficient attention or memory, slow change blindness may unfold because of the nature of the integration mechanisms involved in online perception.

One integration method that would plausibly lead to slow change blindness is a continuous reliance on the recent past—a kind of “active perceptual serial dependence” [5]. Serial dependence is a phenomenon in which observers’ perception of a current stimulus is biased toward recently seen similar stimuli [11–14]. It is characterized by a spatiotemporal integration operator, the continuity field [15], which is agnostic about the level of implementation and could lead to serial dependencies at any or all stages of visual processing. Serial dependence may be consistent with predictive coding and Bayesian optimal theories of visual processing [11, 16], yet could also be heuristic or a result of an interplay between encoding and decoding strategies [11, 17, 18]. Given the correlation of natural world statistics across time [19], using the recent past is computationally efficient and can improve vision during uncertainty [20]: it would make sense for the visual system to employ this strategy during continuous viewing. As serial dependence is modulated by similarity and time, it stands to reason that a series of slight adjustments to an object across time should lead to a series of serially dependent percepts. Across time, these continuously biased percepts could camouflage a slow change [5], leading to slow change blindness.

Only a handful of studies have investigated slow changes, but three provide results hinting at serial dependence. After viewing an image where a face slowly changes expression, observers (unaware of the change) report a facial expression that reflects the face as it had been about 3 s earlier in the morph rather than the actual final facial expression [21]. In another study, observers who viewed a short morph underestimated the age of a face when the preceding morph increased in age and overestimated the age when the preceding morph decreased in age [5], indicating that they were unaware of the extent of the age change. This study did not explicitly ask observers if they noticed the change in age. The authors also used morphs between male and female faces, replicating the dependence of reports on the preceding content [5]. A third study found that observers (blind to the change) who viewed a color-changing object tended to report that the color presented midway through the morph best matched what they had just seen [22]. In each of these studies described, observers’ reports of the final stimulus state were biased toward the past and depended on the preceding stimulus states.

Though these previous studies suggest serial dependence, they cannot make claims about online continuous perception, nor can they rule out other integration methods. To disentangle moment-to-moment perception from morph-long reflective percepts or memories, a handful of other possibilities must be considered. First, observers may simply demonstrate hysteresis: perception is unbiased but has a fixed lag. An observer may report an earlier state simply because their perception has not yet been updated to reflect the most recent stimulus. Second, it is possible that without any awareness of the change, observers calculate and report the average stimulus state when they are prompted to make a judgment about what they have just seen. Temporal ensemble perception could be an example of this [23–25]. Manassi and Whitney (2022) counter this by showing a change in the degree of perceptual bias when the preceding morph traverses the same range via discrete steps instead of continuously, but these videos no longer present the same task to the visual system (the steps may trigger a resetting of reference frame or draw attention to the change). Importantly, all three studies discussed above only probe perception of a static stimulus once after observers have experienced the full task-irrelevant morph; they do not measure perception continuously. This leaves some crucial questions still unanswered: what is the perception of a slowly changing object at each moment in time and how is it generated?

**Fig. 1:**
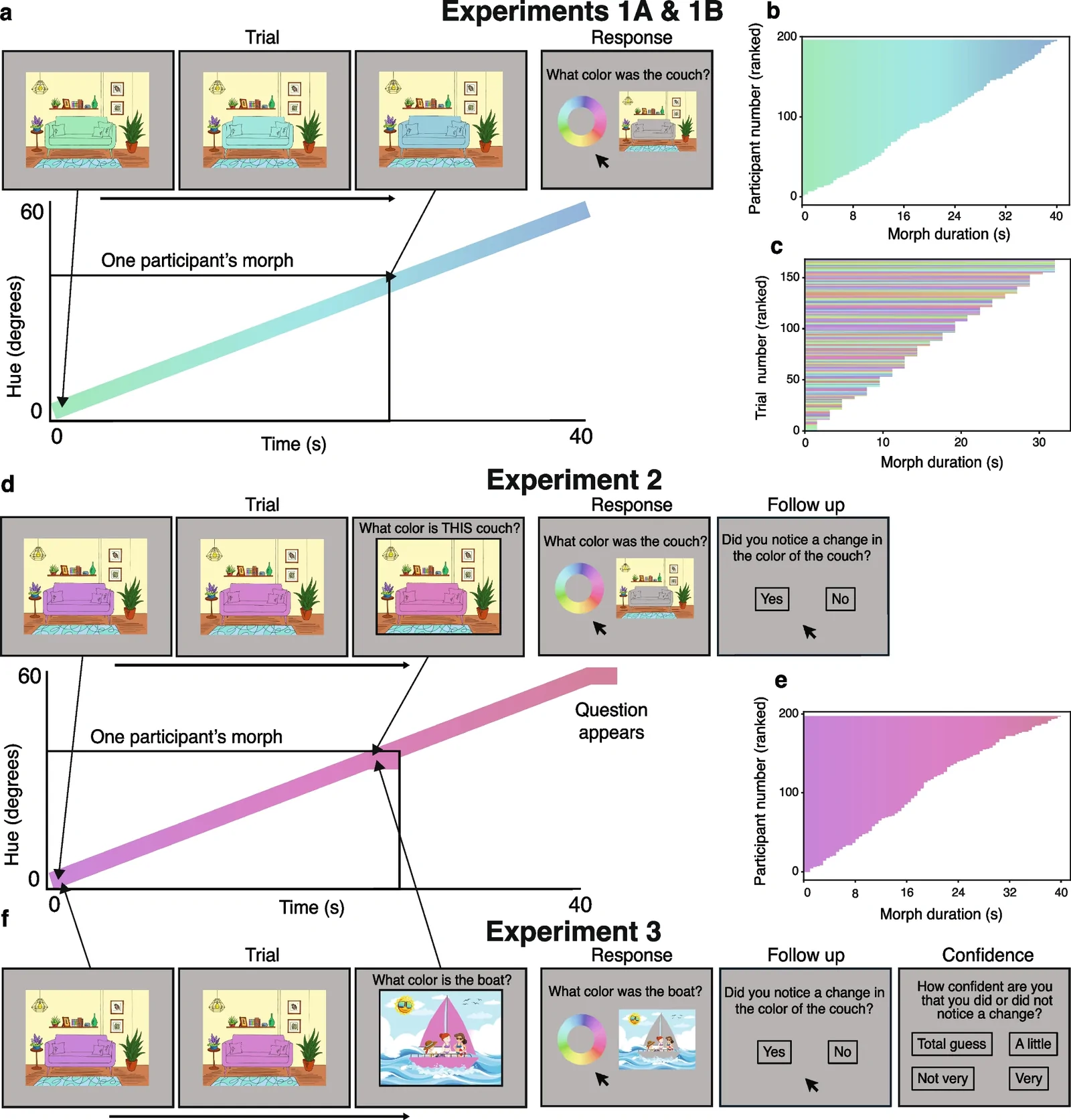
**a** Experiment 1 design. A cartoon couch slowly changed hue through Hue, Saturation, Value (HSV) space at a constant rate across time. On the left, the test couch is shown in its initial state. Below is a plot showing the stimulus motion (hue change) across time. The trial endpoint was randomized for each observer. An example participant’s morph is outlined in black. The rightmost couch reflects the state of the couch just before the participant is asked to make a judgment. After the test couch disappeared, participants were asked to select the couch’s hue from a color wheel using their mouse. As they hovered over the color wheel, the response couch to the right of the color wheel updated to match moused-over hue. Participants clicked to submit their choice. **b** The extent of the morph experienced by each participant in Experiment 1. The x-axis shows the duration of the morph in seconds with the gradient indicating couch color at each moment. On the y-axis is participant, ranked by exposure duration. A single row represents the hue change that a single observer saw, with the rightmost edge of the bar indicating the actual ending hue. Across all observers, the full range of durations and therefore couch colors were sampled. **c** Morph experienced on each trial by an example participant in Experiment 1B. Each participant completed multiple trials and starting hue varied randomly on each trial. The hue changed at a rate of 1 degree per second through Hue-Chroma-Luminance (HCL) space. Trials are ranked by distance. The varying colors of the horizontal bars represent the hue changes in each trial. **d** Experiment 2 design. The question appeared while the couch was still onscreen for the last second of the morph and two additional seconds. After the response, participants were asked whether they noticed a change during the trial. **e** Experiment 2 participants ranked by morph duration. **f** Experiment 3 design. The trial frame on the right shows that the couch has suddenly become a boat at the moment the question appears. Participants made a response about the color of the boat and then reported whether they noticed a change and their confidence in the decision

Here, we rule out hysteresis and averaging and provide support for a role of active perceptual serial dependence in the phenomenon of slow change blindness. In the following experiments, we measure perception at each moment in a morph by accumulating one-shot hue reports from a large number of observers who each viewed a different extent of the total slow hue change. First, we aimed to evaluate the relationship between the extent of the morph experienced and the observers’ reported selected hue (Experiment 1A). Two hundred one observers each experienced a single trial where they viewed a slowly changing colored couch (“test couch”) for a random duration, dictated by the randomly assigned degree of hue change. Participants reported the hue of the couch immediately after the morph ended—making a single hue response by selecting the hue from a color wheel (Fig. 1a). Participants were unaware that the couch could change hue. Because each participant experienced a different amount of preceding hue information prior to entering their color report, the entire range of hue distances relative to the starting point were covered across all participants (Fig. 1b). We predicted that at any moment, observers would report a color that matched an earlier state of the couch (biased toward the past). Next, we conducted Experiment 1B in the lab to evaluate whether perceptual biases could be found on repeated trials. Figure 1c shows the multi-trial structure of Experiment 1b. We hypothesized that the repeated trials would result in a smaller effect size compared to the online one-shot Experiment 1A. In Experiment 2, we aimed to replicate Experiment 1 with a new hue range and further investigate the relationship between slow change blindness and biased perceptual reports. We explicitly asked whether observers noticed a change in the couch color (Fig. 1d), hypothesizing that the majority of observers would be blind to the change. Two hundred one unique participants were exposed to a different and random degree of hue change during a single trial (Fig. 1e). Finally, to explicitly test whether any response bias could be due to serial dependence, we conducted Experiment 3. Observers viewed a slowly changing couch that suddenly became a boat, at the moment of report, while the boat maintained the couch’s planned hue (Fig. 1f). A well-known property of serial dependence is that it is object-selective: when an object changes identity, serial dependence is reduced [14, 26–28]. If serial dependence drives the observer’s response, hue reports to a *different* object should have smaller overall error and minimal bias toward the past. If Experiment 2’s results were response bias or anchoring, there should be no difference between Experiment 2 and Experiment 3. Each of 198 new participants were exposed to a different and random degree of hue change during the single trial (Additional file 1: Fig. S1). Together, the results from these experiments strongly support the idea that active serial dependence underlies slow change blindness.

## Results

### Experiment 1A

**Fig. 2:**
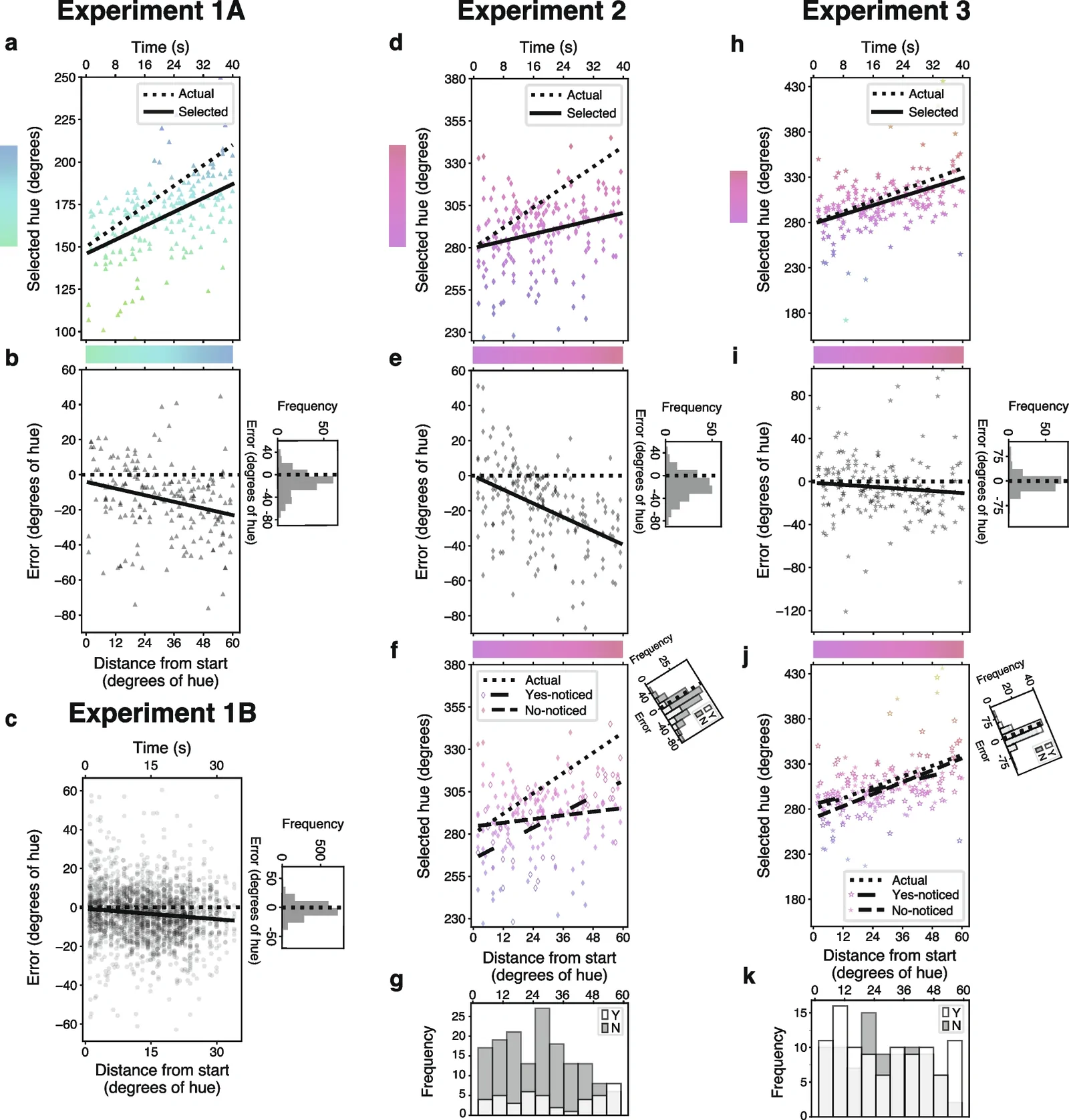
**a** Experiment 1A results (*n* = 197). Selected hue is plotted as a function of the hue distance traveled. Equivalent time is shown on the top x-axis. One triangle is one participant. The x-axis position reflects the relative morph endpoint, and the horizontal gradient bar shows the actual color at each hue distance. The y-axis position, also the color of the triangle, is the selected hue (top) or error (bottom). The dotted black line is a unity line representing ground truth: the actual hue at each distance. Any triangle that does not fall on the dotted line indicates a nonzero error. The solid black line is a regression line fit to the selected hue data. **b** Error is plotted as a function of hue distance from start. The histogram to the right plots the distribution of errors. **c** Experiment 1B results (*n* = 12). Error is plotted as a function of hue distance from start with equivalent time on the top x-axis. Each circle represents one trial. **d** Experiment 2 results (*n* = 198). One diamond is one participant. The dotted black line is a unity line and the solid black line is a regression line fit to the selected hue data. **e** Error is plotted as a function of hue distance from start with equivalent time on the top x-axis. The histogram to the right plots the distribution of errors. **f** Experiment 2 separated by whether the observer reported noticing the couch color change (yes) or not (no). **g** Frequency of noticed response (yes or no) as a function of morph distance. **h** Experiment 3 results (*n* = 185). One star is one participant. The dotted black line is a unity line and the solid black line is a regression line fit to the selected hue data. **i** Error is plotted as a function of hue distance from start with equivalent time on the top x-axis. The histogram to the right plots the distribution of errors. **j** Experiment 3 separated by whether the observer reported noticing the couch color change (yes) or not (no). **k** Frequency of morph distance split by noticed response (yes or no)

Morph duration (time), morph distance (hue), actual ending hue, and selected ending hue were obtained for each participant’s trial. One hundred ninety-seven participants were included for analysis (see Methods for exclusion details). Figure 2a shows selected hue as a function of relative hue distance from start (analogous to time). To evaluate the relationship between hue distance traveled and selected hue, a simple linear regression was fit to the empirical data (selected = 145.9 + 0.684*distance; solid black). The model revealed a moderate, positive, and significant relationship between hue distance traveled and selected hue, *r*(195) = 0.506, *t* = 8.19, *p* < 0.001. That selected hue was predicted by hue distance traveled (analogous to time; Fig. 2a), indicates that observers update their representation across time, and does not simply “stick” with the initial stimulus state (as might be expected if there was anchoring or using “the world as external memory,” O’Regan, 1992). This significant relationship demonstrates that observers do not choose randomly: their choices depend on the preceding morph.

The dotted black line is a unity line representing ground truth: the actual hue at each distance (Fig. 2a). To determine whether the empirical slope differed from the ground truth (slope = 1), we fit a second model including distance, group (0 = ground truth, 1 = empirical), and their interaction. The model yielded a significant distance × group interaction term, confirming that the effect of distance on selected hue differed depending on the group, *t* = − 3.78, *p* < 0.001. Specifically, the slope of the selected hue data was significantly flatter than the slope of the actual hue data. The model also yielded a significant main effect of distance (*t* = 16.9, *p* < 0.001) and a non-significant main effect of group (*t* = − 1.36, *p* = 0.174). A histogram of errors collapsed across the morph, mean of − 14.0 (SD = 20.6), is shown to the right of Fig. 2b. A one-sample *t*-test determined that the mean error was significantly less than zero, *t*(196) = − 9.56, *p* < 0.001. Further, a chi-square goodness-of-fit test confirms that the distribution of errors is significantly different than chance (equal frequency of responses above and below zero), ${\chi }^{2} \left(1, N=193\right)=63.8, p<0.001$: the majority of errors are negative. Together, the significantly flatter empirical slope (0.684) and nonuniform distribution of errors indicate that participants often report an ending hue that is “less than” the true ending hue: a bias toward the past. These results echo previous findings of biased representations after viewing a slowly changing stimulus [5, 21, 22].

To best evaluate how observers’ biases change across time, we examined error (selected hue − actual hue) as a function of morph distance. Figure 2b shows error as a function of hue distance from start. A simple linear regression model revealed a significant negative relationship between hue distance and error (error = − 4.08 + − 0.316*distance): as the endpoint of the morph increased, error became more negative, *r*(195) = − 0.261, *t* = − 3.78, *p* < 0.001. These results provide evidence against hysteresis (a stable lag in observers’ reports) and the implicit updating hypothesis [2], which both predict constant error across the morph (Fig. 2b). Hysteresis tolerates a larger magnitude while implicit updating expects a negative error closer to zero, but both explanations predict a near-zero slope. However, as hue distance traveled increased, observers’ error became significantly more negative (slope = − 0.316; Fig. 2b). The magnitude of the bias in perception (error) depends on the preceding information, with a longer morph exposure producing a greater bias. These results favor an interpretation of serial dependence rather than hysteresis or implicit updating but alone are inconclusive.

### Experiment 1B

The in-lab replication used a color-calibrated monitor and the perceptually uniform color space, Hue-Chroma-Luminance (HCL), to define hue. Morph duration (time), morph distance (hue), actual ending hue, selected ending hue, and starting hue were obtained on each trial. A total of 1924 trials were included for analysis (see Methods for details). A simple linear regression is plotted to visualize the relationship between error and distance (solid black line; Fig. 2c). A linear mixed-effects model with random intercepts for participant and starting hue was fit to evaluate the relationship between error and hue distance. There was a significant negative relationship between distance and error (β= − 0.207, SE = 0.039, *t* = − 5.33, *p* < 0.001), indicating that for each perceptually uniform hue increase in distance from the start, error decreased by 0.2 units. Random-intercept variance was observed across starting hues, *σ*^2^ = 17.6, SD = 4.19, and participants, *σ*^2^ = 3.61, SD = 1.90, with residual trial-level variance of *σ*^2^ = 176, SD = 13.3. The overall mean error was negative (− 3.24, SD = 14.1), which a one-sample *t*-test found was significantly different than zero, *t*(1923) = − 10.0, *p* < 0.001 (histogram to the right of Fig. 2c). A chi-square goodness-of-fit test revealed that the distribution of errors was significantly different than chance,${\chi }^{2} \left(1, N=1899\right)=97.8 , p<0.001$, indicating that observers report an earlier stimulus on a significant majority of trials. The in-lab effect size seemed to be more modest than was found online but given the differences in color spaces between the two studies, a direct comparison might be misleading. Nonetheless, the finding that such biases exist even when observers are knowledgeable about the task and when hue is controlled in a perceptually uniform space. Together, this hints that knowledge about a stimulus’ potential to change does not prevent active perceptual serial dependence from biasing perception.

### Experiment 2

Morph duration (time), morph distance (hue), actual ending hue, selected ending hue, and whether the change was noticed were obtained for each participant’s trial. One hundred ninety-eight participants were included for analysis (see Methods for exclusion details). Figure 2d shows selected hue as a function of relative hue distance from start. A linear regression fit to the empirical data (selected = 279.9 + 0.346*distance; solid black line) revealed that morph distance was a significant predictor of selected hue, *r*(196) = 0.266, *t* = 3.86, *p* < 0.001. This replicated our initial finding that observers’ selected hue depends on hue distance. To evaluate whether the empirical slope differed from ground truth (dotted black line, Fig. 2d), a second model including distance, group (0 = ground truth, 1 = empirical), and their interaction was fit. The model yielded a significant distance × group interaction, confirming that the effect of distance on selected hue differed depending on the group, *t* = − 7.31, *p* < 0.001. Specifically, the slope of the selected hue data was significantly flatter than the slope of the actual hue data. The model also yielded a significant main effect of distance (*t* = 15.8, *p* < 0.001) and a non-significant main effect of group (*t* = − 0.040, *p* = 0.968). A histogram of errors collapsed across the morph, mean of − 18.1 (SD = 22.5), is presented to the right of Fig. 2e. A one-sample *t*-test confirmed that the mean was significantly different than zero, *t*(197) = − 11.3, *p* < 0.001. A chi-square goodness-of-fit test further confirmed that the distribution of errors was significantly different than chance (equal number of responses above and below zero), ${\chi }^{2} \left(1, N=194\right)=87.1, p<0.001$: the majority of errors were negative. Figure 2e shows error as a function of hue distance from start. A linear regression model reveals a significant negative correlation between hue distance and error (error = − 0.114 − 0.655*distance): as the hue distance from start increased, error became more negative, *r*(196) = 0.463, *t* = 7.31, *p* < 0.001. These results also replicate the initial conclusion that observers’ selected hues are reliably biased toward the past. Observers have updated but biased representations across time that are inconsistent with hysteresis or implicit updating. Given the updated final question, we are confident that our results are not due to averaging.

Forty-three out of 198 (21.7%) observers reported noticing the change. Thus, the vast majority of observers experienced change blindness (78.3%) and also reported serially dependent hue judgments. Figure 2f replots the data separated by noticed response. To the right of Fig. 2g is a plot of two histograms overlayed (yes or no noticed) representing the distribution of errors. To assess the relationship between hue distance and selected hue among yes-noticers, a linear regression was fit to the yes-noticed data (selected = 265.2 + 0.770*distance). Given the low number of observers who noticed the change, a two-tailed permutation test was conducted to evaluate the statistical reliability of the empirical slope. Selected hue was permuted 10,000 times and on each iteration the slope was recalculated. The empirical slope was found to be significantly different than the permuted distribution (centered at zero), *p* < 0.001. A model including hue distance, group (0 = ground truth, 1 = empirical), and their interaction was also fit, yielding an interaction term of − 0.231. A two-tailed permutation test found the interaction term to be no different than zero, *p* = 0.249, meaning that the slope of selected hue as a function of distance among yes-noticers was not significantly different than ground truth. This suggests that observers who reported noticing the change have a bias (lower y-intercept) that is stable across time (similar slope).

A separate linear regression was fit to the no-noticed data (selected = 284.5 + 0.181*distance). A two-tailed permutation test was used to evaluate the statistical reliability of the slope. The empirical slope was not reliably different than zero, *p* = 0.087. While selected hue tended to be less than the actual hue, the selected hue did not reliably change with hue distance. A model including hue distance, group (0 = ground truth, 1 = empirical), and their interaction was also fit, yielding an interaction term of − 0.819. A two-tailed permutation test found the interaction term to be reliably different than zero, *p* < 0.001, meaning that the slope of selected hue as a function of distance among no-noticers was significantly flatter than ground truth. This suggests that observers who reported not noticing the change are heavily influenced by the initial couch color.

A Kolmogorov–Smirnov test indicated that the overall distributions of distance in the yes-noticed (*n* = 43) and no-noticed (*n* = 155) were significantly different, *D*(43,155) = 0.250, *p* = 0.025 (Fig. 2g). This indicates that there is a relationship between morph duration experienced and whether an observer reported noticing the change.

### Experiment 3

Morph duration (time), morph distance (hue), actual ending hue, selected ending hue, whether the change was noticed, and observers’ confidence were obtained for each participant’s trial. One hundred eighty-five participants were included for analysis (see Methods for exclusion details). Figure 2h shows selected hue as a function of relative hue distance from start. A linear regression was fit to the empirical data (selected = 278.8 + 0.841*distance; solid black line) which found distance to be a significant predictor of selected hue, *r*(183) = 0.435, *t* = 6.53, *p* < 0.001. To determine whether the empirical slope differed from the unity line (dotted black), an additional model including distance, group (0 = ground truth, 1 = empirical), and their interaction was fit. The model did not yield a significant distance × group interaction, meaning that the effect of distance on selected hue did not differ depending on the group, *t* = − 1.24, *p* = 0.216. This indicates that the slope of the selected hues does not differ from ground truth. In other words, there was no difference between the pattern of observers’ selected hues and the ground truth. The model also yielded a significant main effect of distance (*t* = 11.0, *p* < 0.001) and a non-significant main effect of group (*t* = − 0.286, *p* = 0.775). A histogram of errors collapsed across the morph is presented to the right of Fig. 2i. A one-sample *t*-test found that the mean error (− 5.64, SD = 28.7) is significantly different than zero, *t*(184) = − 2.67, *p* = 0.008. A chi-square goodness-of-fit test revealed that the distribution of errors was significantly different than chance, ${\chi }^{2} \left(1, N=179\right)=12.3, p<0.001$, indicating that observers overall report an earlier stimulus state. Figure 2i shows error as a function of hue distance from start. A linear regression model (error = − 1.19 − 0.159*distance) indicates that this trend is not significant, *r*(185) = 0.089, *t* = − 1.24, *p* = 0.217: there is no significant relationship between reporting error and hue distance traveled.

Ninety-eight out of 185 (53.0%) observers reported noticing the change. Figure 2j replots the data separated by noticed response. To the right is a plot of two histograms overlayed (yes or no noticed) representing the distribution of errors. To assess the relationship between hue distance and selected hue among yes-noticers, a linear regression was fit to the yes-noticed data (selected = 285.4 + 0.662*distance). A permutation analysis found the empirical slope to be reliably different than zero, *p* < 0.001. A second linear regression including hue distance, group (0 = ground truth, 1 = empirical), and their interaction was also fit, yielding an interaction term of − 0.338. A two-tailed permutation test found the interaction term to be no different than zero, *p* = 0.059, meaning that the slope of selected hue as a function of distance among yes-noticers was not significantly different than ground truth. Observers who reported noticing the change (presumably the slow change in the couch) did not show a reliable bias in their hue selections for the new object.

A separate linear regression was fit to only the no-noticed data (selected = 269.9 + 1.11*distance). A two-tailed permutation test found that the empirical slope was significantly different than zero, *p* < 0.001. An additional model including hue distance, group (0 = ground truth, 1 = empirical), and their interaction was also fit, yielding an interaction term of 0.107. A two-tailed permutation test found that the interaction term was not reliably different than zero, *p* = 0.687, meaning that the slope of selected hue as a function of distance among no-noticers was significantly flatter than ground truth. This suggests that when asked to report the color of a new object following a slow change, observers who reported not noticing the slow change do not show a bias in reporting the current color.

A Kolmogorov–Smirnov test indicated that the overall distributions of distance in the yes-noticed (*N* = 98) and no-noticed (*N* = 87) were not significantly different, *D*(98,87) = 0.094, *p* = 0.758 (Fig. 2k), indicating that morph duration was not related to reporting the change. The majority of observers reported that they were highly confident, both those who reported noticing the change and those who reported not noticing it (Additional file 1: Fig. S2). A Kolmogorov–Smirnov test indicated that the overall distributions of confidence responses in the yes-noticed (*N* = 98) and no-noticed (*N* = 87) were not significantly different, *D*(98,87) = 0.177, *p* = 0.097. This indicates that regardless of whether people reported noticing the change or not, they tended to be equally confident in that decision. A chi-square test for independence revealed that there were significantly more observers who reported noticing the change in Experiment 3 compared to Experiment 2,${\chi }^{2} \left(1, N=383\right)=38.8 , p<0.001$.

A chi-square test for independence revealed that there were significantly more observers who reported noticing the change in Experiment 3 compared to Experiment 2,${\chi }^{2} \left(1, N=383\right)=38.8 , p<0.001$. Despite the question asking specifically about a change in the couch, participants in Experiment 3 may have actually reported noticing the change from couch to boat. While purely speculative, this could explain the near doubling of reported noticers. Perhaps the sudden appearance of the boat introduces a temporal or event boundary prompting a re-assessment of object features. If the percept of the couch is biased and the percept of the boat is not, it stands to reason that despite being nearly identical, observers perceive a large change in the hue between the two objects.

### Comparing Experiments 2 and 3

Additional file 1: Figure S3a replots the data from Experiment 2 (diamond, gray) and Experiment 3 on the same plot (star, black), which differed only in the identity of the object at the end of the morph and during report. On the top right is a plot of two histograms overlayed (from each experiment) representing the distribution of errors. A Kolmogorov–Smirnov test found that the overall distributions of error in Experiment 2 (*N* = 198) and Experiment 3 (*N* = 185) were significantly different, *D*(198,185) = 0.207, *p* < 0.001, indicating that error in Experiment 3 was overall smaller and more centered around zero. The empirical slope difference between Experiments 2 and 3 (0.495) was compared to a permuted null distribution formed by permuting experiment labels and recalculating the slope difference 10,000 times. The permutation analysis found that the empirical slope difference was significantly greater than zero, *p* = 0.006 (*α* = 0.05), meaning there is a statistically reliable difference between the slopes of the two experiments. Together, this confirmed that observers’ biased responses in Experiment 2 were specific to the object, rather than hue in general, and therefore driven by serial dependence.

We also compared only the no-noticed observers in Experiments 2 and 3 (Additional file 1: Fig. S3b). A Kolmogorov–Smirnov test found that the overall distributions of error among non-noticers in Experiment 2 (*N* = 155) and Experiment 3 (*N* = 85) were significantly different, *D*(155,85) = 0.184, *p* = 0.038, indicating that even within the non-noticers, error in Experiment 3 was overall smaller and more centered around zero. A permutation analysis found that the empirical slope difference (0.926) among the no-noticers in Experiments 2 and 3 was significantly different than zero, *p* < 0.001 (*α* = 0.05). In other words, the slope of selected hue as a function of hue distance was flatter in Experiment 2. This means that the object change from a couch to a boat significantly impacted observers’ hue selection, specifically when they reported being blind to the slow change. Once again, this shows that the bias is not tied to color but rather the object itself, indicating a role of serial dependence in producing the biases in Experiment 2.

## Discussion

We used independent-observer one-shot trials to evaluate biases in perception of slowly changing objects across time. Consistent with previous work [5, 21, 22], after experiencing a slow change, observers report an object to match an earlier state rather than the true end state (Experiments 1A, 1B, and 2). Expanding on previous work, we further reveal that these perceptual biases exist throughout the slow change, and that the magnitude depends on the preceding content—becoming greater as the current state evolves farther from the initial one (Experiments 1A, 1B, and 2). In Experiment 3, there is a significant reduction in serially biased hue reports when the object changes identity at the end of the morph: confirmation that serial dependence drives the bias in hue reports rather than anchoring, hysteresis, or response bias. Together, these results provide evidence in favor of the active perceptual serial dependence hypothesis [5].

### Comparison between Experiment 1A and Experiment 2

Across two distinct hue ranges and nearly 400 unique participants, Experiments 1A and 2 both provide evidence that perception at any moment while viewing a slow change is serially dependent. The slopes differed slightly between the two experiments, yet small differences in Experiment 2 preclude an analysis of the numerical difference. The hue pairs in each experiment span the same Hue, Saturation, Value (HSV) space “distance” but may not have the same linearity or perceptual distance because absolute color is difficult to control online. The timing of the final question also differed between the experiments. In Experiment 2, the couch remained onscreen when the question appeared, prompting participants to select the ending couch hue and discouraging a report of the average or initial couch hue. The effect was still present despite the static couch hue during the final 2 s, indicating the strength of the bias toward the past. Regardless of the design and slope differences, both experiments revealed the same trend and demonstrate a significant relationship between morph duration and bias indicative of serial dependence.

### Change detection

Intuitively, one might expect that more serial dependence (larger error) leads to slow change blindness while less serial dependence (smaller error) leads to noticing the change. The results in Experiment 2 do tend to support this. However, it is unclear exactly when the yes-noticed observers perceived the change. For example, an observer may notice hue change within 10 s. During the next 30 s, perhaps the observer experiences serial dependence, masking any new changes. At 40 s, the observer reports their biased percept and reports noticing a change. In another case, an observer’s percept is biased yet may be sufficiently different from the starting hue to prompt noticing a difference. Their reported percept is also serially dependent, yet they have overcome the blindness to the change. From the data collected, we cannot distinguish between the two situations. However, the difference in response patterns between the no-noticed and yes-noticed observers is a compelling link between the moment-to-moment percepts suggestive of serial dependence and the overall experience of slow change blindness.

### Continuity fields

These results support the idea that an online stability mechanism, active perceptual serial dependence, smooths perception. For participants who viewed the longest duration of morph (40 s), their reports most closely match the stimulus as it was around 13 s ago (Experiment 1A) and at around 27 s ago (Experiment 2). This suggests that information presented roughly 20 s ago can shape current perception, consistent with estimates of continuity fields in previous research [13]. However, our results alone are insufficient to determine whether the continuity field is determined by time, difference in stimulus features, the rate of change, or a combination of these factors. Further, serial dependence induced slow change blindness may not be complete. For most observers, the smoothing was sufficient to produce slow change blindness. However, successive information that exceeds the bounds of the continuity field may cause observers to notice a change. If the target feature changes too quickly, each successive stimulus state will be different enough to override a stabilization mechanism. Changes which occur more quickly may also introduce a visual transient that triggers detection. Likewise, an object identity change like in Experiment 3 may serve as an event boundary [29, 30] which triggers detection. Observers might also switch from internal to external modes as they accumulate increasingly different information, prompting them to detect the change [31]. Future work should consider evaluating the pattern of error responses across time to stimuli that change at different rates. Why might the visual system strive for continuity at the expense of detecting changes? It is computationally efficient and capitalizes on the overall correlation of natural world statistics over time [19]. It is unlikely that a couch will start slowly changing color. Therefore, using recent perception of the couch color to inform current perception often yields an accurate representation. In other cases, any variation in the color that is not noticed is likely to be due to changes in the surrounding illumination [32]. To interpret these variations as changes in the couch’s feature would be costly, slow, and inefficient. So, is a better strategy to assume stability and miss gradual changes, or assume everything is changing and struggle to adapt to such changes? Our visual system seems to choose the former, at least in some circumstances.

### Value of one-shot experiments

The approach used in our experiments relied on single-observer reports to different morph durations, which, stitched together, sampled an entire morph range. While each observer was completely independent, together their pattern of responses yields a proxy for perception across time. The one-shot approach ensured that participants viewed the slowly changing couch with no knowledge of the end task, no knowledge that the couch could change, and no knowledge of the range of hues the couch traversed. Therefore, there was no contamination of task-goals or expectation, and observers behaved naturally during viewing and response. Repeated trials would risk spoiling the slow change, alter participant strategy during morph viewing, and prevent us from answering our desired question. Nevertheless, Experiment 1B did confirm that the general pattern of results holds even when observers do have prior knowledge of task, design, and stimulus.

### Importance of attention

It is widely agreed upon in the classic change blindness field that attention is necessary for change detection [6, 33]. An explanation of the high rate of slow change blindness might simply be that observers were not paying attention to the stimulus, and instead experienced inattentional blindness [34]. For example, observers who have been watching a seemingly unchanging couch might stop paying attention at some point, and when asked to report the color of the couch, have only a representation from when they were attending to report on. However, the high precision of hue judgments and high confidence ratings suggest that participants did attend to the changing stimulus. Participants knew they needed to continuously attend to the stimulus because they would be asked a question about the stimulus when the trial ended, which would happen randomly but not exceed 40 s in length. Further, observers who do not attend to the changing object should not maintain a representation of it. Therefore, when prompted to report on the final stimulus state in Experiment 2, their attention would be drawn to the current ending state and their report would have little bias. This suggests that inattention would predict a result similar to the implicit updating hypothesis, which is not what was found here. Overall, our results suggest that observers did pay attention.

How is it possible that our observers were attentive, yet still failed to notice the change? Some research has found that attention is not always enough: observers fail to notice changes to the object they are attending and even fixating [35, 36]. Further, serial dependence depends on attention [37–39], with greater attention leading to greater serial dependence [13]. Given that our results provide evidence that slow change blindness arises from serial dependence, it stands to reason that our observers are more blind *because* of their attention, not because of a lack of it. As observers attend to the changing scene, serial dependence works to stabilize the impression, biasing perception and camouflaging the change.

### Importance of memory

Throughout this investigation, we have taken observer reports as a proxy for perception at each moment. However, the report task in Experiment 1A and 1B likely contained some influence of memory. In Experiments 2 and 3, we updated the task to ask observers to make a judgment about a particular stimulus while it was on the screen. While serial dependencies in perception may be considered a form of memory, the updated task in Experiments 2 and 3 greatly reduced the contributions of longer-term memory stores to participants’ responses. Even if we did probe memory here, not only are these results consistent with previous work probing memory during slow change blindness [22], but these results still show robust evidence of serial dependence regardless of whether observer responses include dependencies in memory.

## Conclusions

Integrating information across time is a fundamental task for the visual system. Here, we provide evidence that the visual system uses information from the recent past to generate current perception. This active perceptual serial dependence is an online mechanism that assumes, and in turn promotes, visual stability in features that might actually be changing. Importantly, we demonstrate a connection between serial dependence and slow change blindness by confirming that the majority of our observers were blind to the slow change. Therefore, we propose that the continuously biased percepts generated by active perceptual serial dependence camouflage the slow change may be one avenue to produce slow change blindness.

## Methods

### Participants

In Experiments 1A, 2, and 3, all participants were recruited through Prolific [40] and completed the study online through Pavlovia [41] for pay at the rate of $10/h. Participants were required to use a desktop computer but there was no other exclusion criteria. The study was approved by the University of California, Berkeley Institutional Review Board (IRB) and informed consent was given by each participant before beginning the study. The participants were completely unique across all three experiments. Two hundred one participants completed Experiment 1A, 201 new participants completed Experiment 2, and 198 new participants completed Experiment 3. Experiment 1B was conducted in person. Twelve observers were recruited through the University of California, Berkeley’s Psychology Department Research Participation Program and completed the study for credit (1 credit/h). The study was approved by the University of California, Berkeley IRB and all participants provided written informed consent prior to participation in the study.

### Procedure

The slow changes in these experiments involved a color change of a large, centrally located cartoon couch in a living room scene. Cartoon images were chosen due to their ease in editing, plausible range of colors, and scene-like structure which introduced meaning to the color-changing object. Specifically, hue was changed here because it could be easily manipulated and quantified; each intermediate stage throughout the morph was a valid state (as opposed to a semi-transparent object that would exist during a slow appearance or disappearance) and is a salient feature that is likely to have been in the observers’ awareness despite blindness to the change. Throughout much of the manuscript, this slow color change is be referred to as a hue change because this was the aspect of color that was changed.

In the online experiments (1A, 2, and 3), each participant experienced a single trial where they viewed the colored couch (“test couch”) for a random duration, dictated by the randomly assigned degree of hue change. Timing of the morph took into account the device’s frame rates to ensure accurate presentation. Participants reported the hue of the couch immediately after the morph ended—making a single hue response (Fig. 1). Participants were unaware that the couch could change hue. Before beginning, participants were given instructions.

In Experiment 1A, the instructions read: “You’ll see a colored image of a couch in a living room for a random duration, up to 40 s. After that, you’ll be asked to report the color of the couch. Try to be as accurate as possible! When you are ready, press ‘space’.” Therefore, though observers did not know that the couch could change in hue, they did know that they would be asked to report on the color of the couch they viewed. After the couch image disappeared from the screen, a color wheel and corresponding “response couch” (the same gray as the monitor background) were presented. Participants were given the following instructions: “What color was the couch? Hover over the color wheel to adjust the color of the couch. When you have reached your choice, click with the mouse.” As the participants hovered over the wheel, the center of the color wheel and the “response couch” were updated to reflect the current color under their mouse. This feature was included to account for any simultaneous-color-contrast effects resulting from the other colors present in the scene and to ensure as accurate a response as possible. The “test couch” always began with a hue of 150 degrees and increased at a rate of 1.5 degrees/s for up to 40 s (Fig. 1b). The color wheel was defined in HSV space and contained all hues (0–359 degrees) at a saturation of 0.40 and value of 0.82. The color wheel contained more colors than the participant was exposed to during the trial, but all colors that the participant saw were contained in the color wheel. The orientation of the color wheel (which color was at the top) was randomized for each participant. Participants selected their choice by clicking with the mouse.

In Experiment 2, the instructions were altered slightly to better align with the updated test question. The new instructions read: “You’ll see a colored image of a couch in a living room for a random duration, up to 40 s. Please pay attention to the couch. At some point a black outline will surround the image and you will be asked a question about the couch. Try to be as accurate as possible! When you are ready, press ‘space’.” As in Experiment 1A, the “test couch” was shown on the screen for a random duration, dictated by the randomly assigned degree of hue change and each participant completed only a single trial. For the last second of the morph (1.5 degrees of hue change) and two additional seconds of static hue (the ending hue), a black outline box and the words “What is the color of THIS couch?” appeared on the screen (Fig. 1d). This question was intended to prompt participants to specifically report the color of the couch in front of them rather than reporting an average from across the morph or any earlier state, reducing the contributions of memory. The “test couch” and question then disappeared, and the color wheel, “response couch,” and text were presented to collect the color judgment. After the participant submitted their color choice, they were then asked: “While viewing the couch on the screen, did you notice any change in the color of the couch?” and responded by clicking a “yes” box or a “no” box on the screen.

In Experiment 3, the instructions were altered slightly to better align with the updated test question. The new instructions read: “You’ll see a colored image of a scene for a random duration, up to 40 s. Please pay attention to the image. At some point a black outline will surround the image and you will be asked a question about the scene. Try to be as accurate as possible! When you are ready, press ‘space’.” The trial began with the slowly changing “test couch” (Fig. 1f). At a pre-determined random moment, the couch image was changed immediately to an image of a boat (“test boat”) where the boat hue maintained the planned couch hue. For the last second of the morph (1.5 degrees of hue change) and two additional seconds of static hue, observers viewed the boat instead of the couch. A black outline box and the words “What is the color of the boat?” appeared on the screen for these 3 s. The boat and question then disappeared, and the color wheel and text with a “response boat” were presented to collect the color judgment. Participants used the color wheel to report on the color of the boat rather than the color of the couch. After the participant submitted their color choice, they were then asked: “While viewing the couch on the screen, did you notice any change in the color of the couch?” and responded by clicking a “yes” box or a “no” box on the screen. Participants were then asked about the confidence of their answer, choosing “Total guess,” “Not very confident,” “A little confident,” or “Very confident” (coded as 1, 2, 3, 4). Experiment 3 was identical to Experiment 2 except for the abrupt change the boat and corresponding boat-color question.

The procedure for Experiment 1B closely followed the procedure from Experiment 1A, but a few changes were made to account for the repeated trials. The 12 observers completed an average of 195 trials each. Eight participants completed all 200 trials. Four participants completed 179, 199, 181, and 181 trials, respectively. The experiment was designed so that each trial had a 1 in 6 chance (16.67%) of not changing. This was to introduce uncertainty and ensure that participants were paying attention throughout the morph and were not included in analysis. Colors were defined in the perceptually uniform HCL space with a constant chromaticity [35] and lightness (70). The starting hue was chosen randomly and the distance varied randomly on each trial and images were presented on a gamma-corrected and color-calibrated monitor (Fig. 1c). The hue changed at a rate of 1 degree per second for all trials (this is slower than the online studies and was chosen due to the nature of the repeated task). Each trial began with the image of the couch on the screen. The couch remained on the screen for a random duration and may or may not have changed hue. After the couch was removed from the screen, participants were asked to use their mouse to select the hue that best matched the couch they had just seen. The orientation of the color wheel was randomized on each trial. Once the participant submitted their response, there was a brief 100-ms delay and the next trial began. The task was the same for each trial. As such, participants knew from trial 2 onward that they would explicitly be asked about the color of the couch. Further, the participants knew that the couch color could change. Aside from the slight stimulus modifications and the nature of the multi-trial design, the in-lab experiment was identical to Experiment 1A.

### Preprocessing—online one-shot data

In the online experiments, clicking the center of the color wheel (gray) progressed the experiment despite being an invalid color choice. Observers who selected an invalid color were excluded. Three observers were excluded in Experiment 1A, 1 in Experiment 2, and 6 in Experiment 3.

Participants were asked to report whether they had normal or corrected-to-normal (wearing glasses or contacts) vision, and whether they were colorblind. These responses were used to split participants into an “atypical vision” and a “normal vision” group (Table 1). In Experiment 1A, the “atypical vision” group included any observer who self-reported colorblindness, self-reported “no” to having normal vision, as well as observers who did not respond to either question (due to a bug that allowed participants to continue without making a selection), “unknown status” observers. More specifically, 4 observers out of 198 (~ 2%) self-reported colorblindness and 4 observers (2%) did not respond to the question. This percentage is not far from the reported global incidence. Thirty-three observers self-reported “no” to the question about normal vision (~ 17%) and 1 observer did not respond (< 1%). Two observers reported both colorblindness and non-normal vision. These proportions are high but include observers who did not respond to the questions. A two-tailed permutation analysis was conducted to determine whether the difference in mean error between the “atypical vision/unknown status” (*N* = 39) and “normal vision” (*N* = 159) groups was significant. The empirical mean difference (− 1.00) was compared to a permuted null distribution, testing the null hypothesis that there is no difference between the means. To create the null distribution, the group labels were randomly permuted 10,000 times and the mean difference was recalculated at each permutation. The resulting *p* value was 0.807 (*α* = 0.0167), indicating that the empirical mean error difference was not significantly different than zero. Therefore, no data was excluded based on self-reports during the analyses.

**Table 1:** The response counts to questions about normal vision and color blindness in the online experiments (1A, 2, and 3)

		Yes	No	No response
Experiment 1A	Color blind	4	190	4
	Normal vision	164	33	1
Experiment 2	Color blind	6	193	1
	Normal vision	150	50	0
Experiment 3	Color blind	4	188	0
	Normal vision	143	46	3

In Experiment 2, the “atypical vision” group included any observer who self-reported colorblindness, self-reported “no” to having normal vision, as well as observers who did not respond to either question, “unknown status” observers. Six out of 200 observers (3%) self-reported colorblindness and 1 observer (< 1%) did not respond to the question. Fifty observers self-reported “no” to the question about normal vision (25%). One observer reported both colorblindness and non-normal vision. A permutation analysis was conducted to determine whether the difference in mean error between the “atypical vision/unknown status” (*N* = 55) and “normal vision” (*N* = 145) groups was significant. The empirical mean difference (1.77) was not significantly different than zero, *p* = 0.652 (*α* = 0.0167). Therefore, no data was excluded based on self-reported vision during the analyses.

In Experiment 3, the “atypical vision” group included any observer who self-reported colorblindness or self-reported “no” to having normal vision. Four out of 192 observers (2%) self-reported colorblindness. Forty-six observers self-reported “no” to the question about normal vision (25%) and 3 observers did not respond (1.5%). No observers reported both colorblindness and non-normal vision. A permutation analysis was conducted to determine whether the difference in mean error between the “atypical vision” (*N* = 53) and “normal vision” (*N* = 139) groups was significant. The empirical mean difference (− 9.89) was not significantly different than zero, *p* = 0.112 (*α* = 0.0167). Therefore, no data was excluded based on self-reported vision during the analyses.

For each participant, error was calculated as selected ending hue − actual ending hue. Raw errors were wrapped to the range [− 180, + 180] where errors less than − 180 became 360 + error and errors greater than 180 became error − 360. In each experiment, participants with an error more than three times the standard deviation away from the mean were excluded also from analysis and are not depicted in any figures (inclusion or exclusion of these participants does not meaningfully change the results). One additional observer was excluded in Experiment 1A, 2 additional observers were excluded in Experiment 2, and 7 additional observers were excluded in Experiment 3. Selected ending hue was recalculated by adding error to the actual ending hue. This effectively wrapped and centered selected ending hue.

### Preprocessing—in-lab multi-trial data

There were no trials excluded in Experiment 1B due to invalid colors. Error was calculated as selected ending hue − actual ending hue and wrapped to [− 180, 180]. Trials with an error greater than three times the standard deviation above the group mean or less than three times the standard deviation below the group mean (errors less than − 68.25 or greater than 62.52 degrees) were excluded from analysis and are not depicted in any Figs. (36 trials, ranging from 0 to 13 trials per participant, average of 3 trials per participant).

### Analysis

Given that hue space is circular, both the absolute hue reports and the error came from a von Mises distribution. However, the range of the circle that the stimulus covers was 60 degrees (1/6 of the circle) and the standard deviation of errors was between 14 and 28 degrees for all four experiments. As a result, the data within the narrow range covered in these studies were effectively linear: there was no wrapping. Therefore, the data met the assumptions of a linear regression, which was easier to visualize, explain, and interpret. To verify that this choice was appropriate, the data was re-analyzed a von Mises regression and replicated the initial results.

In Experiment 1B, a linear mixed-effects model with distance as a fixed effect and random effects of participant and starting hue was used to predict relative selected hue. Random slopes for participant and starting hue explained very little variance (0.01 and 0.004, respectively), and the model suffered from nonconvergence. Therefore, the decision was made to drop random slopes and just include random intercepts in the analyses.

## Supplementary Information

Additional file 1: Figures S1–S3. Fig. S1 The extent of the morph experienced by each participant in Experiment 3. Fig. S2 Frequency of confidence ratings split by noticed response in Experiment 3. Fig. S3 The results from Experiments 2 and 3 replotted on the same graph.

## Data Availability

All data generated or analyzed during this study are included in this published article, its supplementary information files and publicly available repositories. Data and analysis scripts are available at https://osf.io/wdqp8/. [42].

## Abbreviations

IRB: Institutional Review Board
HSV: Hue, Saturation, Value
HCL: Hue-Chroma-Luminance
