# Supplementary material for: Slow change blindness from serial dependence: slowly changing objects are perceived as biased toward the past
Source: BMC Biol. 2026 Aug 3;24:225. doi: 10.1186/s12915-026-02700-w (PMC13729324; doi:10.1186/s12915-026-02700-w)
Supplement: Supplementary file 1 [file 12915_2026_2700_MOESM1_ESM.docx]

**Supplementary Figures**

*Supplementary Figure 1. The extent of the morph experienced by each participant in Experiment 3. The x-axis shows the duration of the morph in seconds with the gradient indicating couch color at each moment. On the y-axis is participant, ranked by exposure duration. A single row represents the hue change that a single observer saw, with the rightmost edge of the bar indicating the actual ending hue. Across all observers, the full range of durations and therefore couch colors were sampled.*

*Supplementary Figure 2. Frequency of confidence ratings split by noticed response.*

*Supplementary Figure 3. The results from Experiments 2 (diamond, gray) and 3 (star, black).* ***a*** *All observers from the two experiments (combining Figures 2d and 2h).* ***b*** *Only the no-noticed observers from Experiments 2 and 3 (combining Figures 2f and 2j).*
